# Synthetic Emotions and the Illusion of Measurement: A Conceptual Review and Critique of Measurement Paradigms in Affective Science

**DOI:** 10.3390/brainsci15090909

**Published:** 2025-08-23

**Authors:** Dana Rad, Corina Costache-Colareza, Ruxandra-Victoria Paraschiv, Liviu Gavrila-Ardelean

**Affiliations:** 1 Centre of Research Development and Innovation in Psychology, Faculty of Educational Sciences, Aurel Vlaicu University of Arad, 310032 Arad, Romania; dana@xhouse.ro; 2 Facultatea de Știintele Educației, Comunicare și Relații Internaționale, Universitatea Titu Maiorescu, 040051 Bucharest, Romania; 3 Facultatea de Psihologie, Universitatea Titu Maiorescu, 040051 Bucharest, Romania; 4 Prosthetic Dentistry, Faculty of Dental Medicine, “Vasile Goldiș” Western University of Arad, 310025 Arad, Romania; liviugav@yahoo.com

**Keywords:** synthetic emotion, natural emotion, emotion measurement, affective neuroscience, affective computing, emotional authenticity, neurodiversity, polygraphy, emotion modeling, ecological validity

## Abstract

The scientific study of emotion remains fraught with conceptual ambiguity, methodological limitations, and epistemological blind spots. This theoretical paper argues that existing paradigms frequently capture synthetic rather than natural emotional states—those shaped by social expectations, cognitive scripting, and performance under observation. We propose a conceptual framework that distinguishes natural emotion—spontaneous, embodied, and interoceptively grounded—from synthetic forms that are adaptive, context-driven, and often unconsciously rehearsed. These reactions often involve emotional scripts rather than genuine, spontaneous affective experiences. Drawing on insights from affective neuroscience, psychological measurement, artificial intelligence, and neurodiversity, we examine how widely used tools such as EEG, polygraphy, and self-report instruments may capture emotional conformity rather than authenticity. We further explore how affective AI systems trained on socially filtered datasets risk replicating emotional performance rather than emotional truth. By recognizing neurodivergent expression as a potential site of emotional transparency, we challenge dominant models of emotional normalcy and propose a five-step agenda for reorienting emotion research toward authenticity, ecological validity, and inclusivity. This post-synthetic framework invites a redefinition of emotion that is conceptually rigorous, methodologically nuanced, and ethically inclusive of human affective diversity.

## 1. Introduction

Emotion science stands at a critical crossroads. Despite decades of inquiry across psychology, neuroscience, and cognitive science, the concept of “emotion” remains fundamentally elusive. Researchers continue to disagree on whether emotions are biologically hardwired responses, social constructions, subjective feelings, or predictive brain states [1]. This conceptual vagueness creates not only theoretical ambiguity but also practical inconsistencies in measurement, diagnosis, and interpretation [2]. At the heart of this crisis lies a question too rarely asked: are we truly measuring natural emotional phenomena—or are we systematically capturing synthetic, distorted, or contextually manufactured responses?

The distinction between natural and synthetic emotion forms the central thesis of this paper. Natural emotions are understood here as spontaneous, biologically grounded, and context-responsive states. In contrast, synthetic emotions are those shaped or constructed by social norms, performance expectations, cultural scripts, or the very act of observation. When individuals are aware of being evaluated—by researchers, machines, peers, or themselves—their emotional responses often shift to fit perceived expectations [3]. This observer effect, well-established in behavioral science, applies equally to affective expression, raising serious questions about the ecological validity of our emotion assessments [4]. We use the term synthetic not to imply falseness, but to denote socially constructed, context-driven emotional expressions shaped by expectations.

Furthermore, emotion research often conflates three distinct constructs: affect, emotion, and feeling. Affect refers to raw, physiological arousal; emotion describes the cognitive, behavioral, and social interpretation of affect; feeling represents the subjective awareness of this state [5]. Without clear differentiation, instruments and paradigms designed to assess “emotion” may unintentionally capture only fragments or surface-level simulations of the emotional experience. As a result, current models may privilege synthetic over natural responses, leading to distorted theories of personality, cognition, and behavior [6].

The problem intensifies when considering the influence of standardization. Most psychological assessments—self-reports, EEG readings, facial expression coding—rely on structured procedures that presuppose stable and replicable emotional patterns [7]. Yet, by enforcing normative conditions, these methods risk eliciting predictable but inauthentic emotional states. Emotion, under such constraints, becomes less an emergent phenomenon and more a rehearsed performance [8].

Synthetic emotions, then, are not “fake” in the sense of being consciously deceptive but are “manufactured” through adaptive mechanisms. Social desirability, emotional labor, impression management, and trauma-based masking all contribute to a cumulative system of emotional simulation [9]. These responses are learned, reinforced, and internalized until even the individual may be unaware of their divergence from natural affective experience [10].

The rise of artificial intelligence and human–computer interaction further complicates the picture. As we teach machines to detect, simulate, or generate emotion, we confront a paradox: we train algorithms on datasets drawn from human responses that are themselves deeply synthetic [11]. Emotional AI may thus be learning not what humans feel, but what they believe they should express [12].

Moreover, polygraph tests, affective computing systems, and EEG-based emotion recognition tools often presuppose a one-to-one mapping between physiological signals and emotional authenticity. Yet emotional simulation—whether conscious or unconscious—can trigger similar somatic signatures as genuine affect [13]. This calls into question the foundational assumption that emotional truth is detectable through surface signals alone [14].

Beyond technology, this framework helps us re-examine social and clinical phenomena. Shame, for example, is rarely a direct response to threat or injury; it emerges through complex processes of internalization, identity development, and social comparison. It may be more accurate to view shame as a synthetic rather than natural emotion—learned over time, reinforced through culture, and often maladaptive [15]. Similarly, many expressions of empathy, outrage, or even joy in public or digital spaces may function more as emotional performances than true affective states [16].

This synthetic emotional scaffolding may be even more visible—or conversely, less accessible—in neurodivergent populations. Individuals on the autism spectrum, for example, often express emotion in ways that diverge from social norms and are frequently mischaracterized as lacking affect [17]. In reality, they may be expressing natural emotion more transparently, precisely because they do not default to the culturally learned scripts that shape synthetic responses or constructed emotional performances [18].

The implications are profound: if our science is based on systematically studying synthetic emotion, our theories of human behavior may be describing something artificial. What we label as “normal” may be an adaptation to social expectations, not a reflection of authentic emotional architecture [19]. This challenges the epistemological foundations of affective science and necessitates a rethinking of both theory and method.

In this paper, we propose a new interpretive lens: synthetic vs. natural emotion. We suggest that much of what we have been studying as “emotion” may be better understood as emotionally adaptive behavior shaped by context, cognition, and the pressure of being seen [20]. To address this, we must critically evaluate our tools, clarify our definitions, and develop new methods that allow natural emotion to emerge unencumbered. Only by doing so can emotion science move beyond the illusion of measurement and toward the reality of human feeling [21].

Through a critical integration of interdisciplinary findings, this paper aims to reframe foundational assumptions about emotional authenticity, measurement validity, and the nature of synthetic affect. The resulting framework contributes to ongoing debates by clarifying conceptual boundaries and proposing a typology that reflects the complexity of lived emotional experience.

This paper adopts a critical and interdisciplinary approach, grounded in the premise that dominant methods in affective science risk conflating emotional performance with emotional authenticity. Such conflation has significant implications, particularly in applied contexts such as education and digital media, where synthetic emotional norms—such as “expected” expressions of empathy, positivity, or resilience—may influence behavior, engagement, and psychological wellbeing. By deconstructing these norms and their technological scaffolding, we aim to foster a more inclusive and ecologically valid understanding of emotion.

Thus, this paper adopts a critical–conceptual stance to address foundational assumptions within affective science and the wider application of emotional measurement. Our approach is driven by the recognition that current methods often equate observable behavior or physiological signals with internal emotional states, thereby neglecting the complex social, contextual, and constructed dimensions of emotion. The central objective is threefold: (1) to introduce a nuanced typology of emotion that reflects the interplay between authenticity, performance, and persistence; (2) to critically evaluate the implicit assumptions underlying measurement tools in psychology, neuroscience, and computational systems; and (3) to explore the implications of synthetic emotional constructs in educational, technological, and media contexts.

Empirical evidence supports this critique. Barrett and collaborators [22] highlight how facial expressions are shaped by cultural and situational variables, undermining the notion that emotions can be directly inferred from facial movement alone. Kosilo and collaborators [23] demonstrate that authenticity is a cognitively and neurally distinct dimension, with individuals exhibiting different brain responses to genuine versus posed expressions of laughter and crying. Furthermore, Zhang and collaborators [24] argue that EEG-based emotion recognition systems struggle with ecological validity and context sensitivity, often failing to distinguish between genuine affective states and surface-level emotional cues. Together, these findings justify a critical reassessment of how emotional authenticity is conceptualized, measured, and applied in both scientific inquiry and real-world settings.

## 2. Conceptual Framework: Natural vs. Synthetic Emotion

This paper addresses a growing epistemological and methodological concern in affective science: the tendency to equate observable emotional behavior with emotional authenticity. The central research problem concerns how widely used tools and paradigms might unintentionally reinforce synthetic emotional expressions—those shaped by external norms and strategic modulation—as normative. The guiding objective is to critically examine the conceptual and methodological boundaries between natural and synthetic emotion. Through a theoretical–analytical approach, we synthesize insights from neuroscience, psychology, and AI studies to offer a revised emotion typology and propose an agenda for rethinking emotional measurement.

A rigorous understanding of emotion depends on conceptual clarity. Without clearly defined boundaries between natural and synthetic emotional constructs, researchers may conflate adaptive performances with authentic affective states. We distinguish natural from synthetic emotion, outline intermediary states, and propose a typology aligned with affective complexity.

### 2.1. Natural Emotions: Evolutionary Origins and Spontaneity

Natural emotions are often described as biologically ingrained responses to stimuli essential for survival. These include rapid, spontaneous reactions such as fear, anger, or disgust, which are accompanied by characteristic physiological and expressive patterns [25]. Such emotions are believed to have evolutionary continuity and are commonly found across cultures and even species [26]. They arise independently of reflective thought or deliberate intention and tend to be time-bound, stimulus-specific, and self-organizing in nature [27].

Importantly, natural emotions are typically interoceptively driven—emerging from bodily states rather than external appraisals [28]. Aesthetic awe, maternal bonding, or grief in response to loss are examples of emotional phenomena that appear to bypass social scripting or cognitive mediation [29]. These emotions often arise in private moments or natural environments, where performance pressures are minimized and the individual is not under scrutiny [30,31].

Natural emotion may thus be defined by three key features: (1) spontaneity—not elicited through instruction or expectation; (2) embodiment—arising from the internal milieu of the body; and (3) minimal social distortion—expressed without concern for external judgment. These criteria serve as a baseline against which synthetic emotion can be evaluated.

While natural emotion is primarily associated with interoceptively driven, bottom-up signals originating from the body (e.g., viscera, sensorimotor systems), the boundary between these and top-down, cognitively modulated emotions is complex and often blurred in empirical settings. Rather than assuming a binary division, we propose a relative continuum based on dominance and temporal sequencing of inputs. Evidence from affective neuroscience suggests that early physiological responses—such as heart rate variability, skin conductance, and activity in the anterior insula and brainstem—often precede and shape conscious appraisal [32,33]. Thus, the timing of affective onset and its neural pathways may serve as indirect indicators of interoceptive origins. While current tools may not permit absolute verification of an emotion’s source, we advocate for a layered interpretive framework that considers reflexivity, context, and the performative demands of the situation. This approach underscores the need for epistemic humility when interpreting measured emotion and shifts the focus from classification to critical contextualization.

### 2.2. Synthetic Emotions: Cultural Scripts and Emotional Simulation

In contrast to natural emotional experiences that arise spontaneously from internal states or immediate environmental stimuli, synthetic emotions are constructed, rehearsed, or strategically regulated in response to external cues, societal expectations, or performance demands. These emotions are not purely fabricated but are shaped by internalized roles, culturally embedded norms, interpersonal scripts, and self-preservative mechanisms [34]. A smile delivered in customer service, the suppression of fear in high-pressure work environments, or performative sorrow at socially scripted events like funerals illustrate how emotions may be regulated more for others than for the self [35].

Synthetic emotions or strategically constructed emotional displays are not necessarily insincere in the moral sense; rather, they are manufactured through repeated social learning, unconscious rehearsal, and adaptive behavior. From an early age, individuals are socialized into emotional conformity—taught which emotions to express, modulate, or conceal—based on dimensions such as gender, social class, cultural identity, or neurocognitive style [36,37]. With time, these externally shaped affective patterns can become embedded as habitual emotional responses, diverging from the individual’s spontaneous, natural affective baseline.

Evidence increasingly suggests that such synthetic emotions may dominate the affective landscape of modern life, particularly in contexts where emotional self-management has become a normative expectation. In contemporary society, emotional labor is not confined to specific professions—it has become a generalized social skill. Emotional expression is often governed by strategic self-presentation, tailored to achieve social acceptance, career progression, or online visibility [38,39].

This phenomenon is particularly visible in digital environments. Social media platforms often prioritize and reward specific emotional expressions—enthusiasm, positivity, gratitude—while discouraging or penalizing vulnerability, ambivalence, or sadness. The result is a pervasive emotional curation that favors synthetic positivity over authentic complexity. Users learn to perform emotions that align with platform norms, sometimes amplifying feelings they do not fully experience or suppressing those that feel too risky to reveal. Similarly, in professional and academic settings, emotional suppression, tone regulation, and strategic smiling are common, expected, and often rewarded behaviors.

Such dynamics reflect the broader concept of emotional management, which encompasses both effortful emotion regulation—modifying one’s internal state to match situational demands—and strategic emotional expression—choosing how to outwardly display emotions to produce a specific interpersonal effect. While emotional intelligence is often celebrated as a positive attribute, it can also reinforce the primacy of control over unfiltered emotion, leading individuals to favor regulation over honest emotional experience.

Synthetic emotions thus emerge as a byproduct of adaptation to systems that reward emotional predictability, consistency, and appropriateness. But this adaptation comes with psychological costs. Emotional masking, for instance, is commonly employed by both neurotypical and neurodivergent individuals to manage daily social or institutional demands [40]. Though often necessary, such masking can result in affective dissonance—a mismatch between internal feeling and external expression—and may ultimately contribute to identity fragmentation, burnout, or emotional fatigue [41].

Thus, synthetic emotions or strategically constructed emotional display exhibit three defining features:

- Context-dependence—they emerge primarily in response to professional, institutional, or social roles.
- Cognitive mediation—they involve deliberate internal monitoring and adjustment.
- Goal-orientation—they serve interpersonal, reputational, or self-regulatory functions.

Recognizing the extent to which synthetic emotions pervade modern life forces us to rethink the nature of authenticity in emotional expression. In environments shaped by surveillance, social media, or formal hierarchies, what we commonly observe—and measure—may not be natural emotional states, but rather adaptive performances shaped by collective scripts. While this emotional simulation is often necessary for social cohesion and survival, it also highlights the urgent need to distinguish between surface expression and genuine affective experience in both research and human interaction.

### 2.3. Between the Lines: Hybrid, Masked, and Residual Emotion

Emotional life rarely conforms to binary categories. While the distinction between natural and synthetic emotions offers a useful conceptual anchor, lived emotional experience often unfolds in more ambiguous, fluid, and layered ways. In reality, many emotions exist in transitional or composite states, occupying spaces between spontaneous internal responses and socially shaped external displays. Recognizing this ambiguity is essential for building models of emotion that reflect the complex and dynamic nature of human affective experience.

Hybrid emotions occur when internal affective states are partially shaped or intentionally modulated by external goals or interpersonal dynamics. These emotions are neither fully natural nor fully synthetic but involve a blending of authenticity and strategic expression. For example, an individual may feel genuine sadness but enhance its visibility in order to elicit empathy, signal vulnerability, or reinforce relational bonds [42,43]. This emotional amplification may be intentional or unconscious, but in either case, it reflects the interplay between felt emotion and social utility. Such hybrid emotions often emerge in high stakes settings—like caregiving or therapy—where authenticity is blended with emotional strategy.

Residual emotions represent a distinct form of complexity—affective states that linger beyond immediate contexts, shaped more by internal processes such as memory, cognitive encoding, or identity structures [44]. For instance, a person may continue to feel shame, longing, or grief months or even years after the originating event. These residual emotions are not always accessible to conscious reflection; they may arise suddenly in the form of physiological tension, mood shifts, or behavioral impulses, indicating the brain’s long-term encoding of emotional episodes. In computational terms, one could think of residual emotions as low-frequency background processes—active yet not always foregrounded—that influence decision-making, social interpretation, and self-concept over time.

Masked emotions add yet another layer of complexity. These involve natural emotional responses that are consciously or unconsciously suppressed, redirected, or replaced in the act of expression. Common among trauma survivors and neurodivergent individuals, masked emotions reflect a fundamental adaptive strategy: to protect the self by concealing vulnerability or deviation from social norms [45]. This suppression may become habitual, leading individuals to develop alternative emotional signatures—what might appear as emotional flatness, irritability, or disconnection. When the distance between felt emotion and its external expression becomes too wide or chronic, the individual may experience identity fragmentation, emotional dysregulation, or psychosomatic manifestations [46]. This emotional latency is often misunderstood in clinical and social contexts, where expression is equated with experience and silence is misread as absence.

Given the multifaceted nature of these emotional forms, a more refined typology of emotion is necessary—one that captures the fluid, stratified, and sometimes contradictory ways in which emotion is lived and displayed. The following five categories are proposed:

- Transparent emotion (directly felt and expressed): emotion that is both felt and directly expressed, free from distortion or modulation.
- Camouflaged emotion (concealed from external expression): emotion that is felt internally but intentionally or reflexively concealed.
- Synthetic emotion (strategically constructed): emotion that is strategically constructed for social, relational, or professional purposes.
- Contaminated emotion (distorted over time by social or internal pressures): emotion that begins as natural but becomes distorted through cumulative social feedback, repeated suppression, or internalized trauma.
- Residual emotion (emotionally persistent beyond context): emotion that persists after the originating stimulus, often sustained by memory, identity narratives, or embodied neural patterns [47,48].

For a summary of these five types, see Table 1.

This typology integrates subjective and computational understandings, recognizing emotion as both felt experience and encoded signal. For psychologists, it offers a lens through which to better understand emotion in therapy, diagnosis, and everyday functioning. For computer scientists and engineers working on affective systems, it provides a layered architecture for modeling emotional complexity beyond binary or categorical outputs.

This expanded framework has practical implications. It cautions against reductive mappings between emotional input and behavioral output, as is common in facial recognition systems and affective computing platforms. It also informs the development of psychometric instruments and clinical protocols that account for discrepancies between reported and felt emotion. By acknowledging transitional states—such as hybrid, masked, and residual emotions—we move closer to a theory of emotion that reflects how human beings actually feel, adapt, and relate.

Recognizing the distinction between natural and synthetic emotion reframes major debates in psychology. Basic emotion theory and social constructivist approaches are no longer mutually exclusive—they may simply describe different ends of the same continuum [49,50]. Natural emotions could represent evolutionarily grounded templates, while synthetic emotions arise from learned regulatory and expressive strategies.

This framework demands that we reevaluate existing research instruments. If most self-report scales, facial recognition tools, or EEG paradigms are designed in contexts that prompt synthetic performance, they may be measuring compliance, role adherence, or perceived appropriateness—not raw emotional truth.

Understanding emotion as a fluid interaction between natural embodiment and synthetic adaptation allows for a more accurate, inclusive, and ethically sensitive model of human affect. It opens pathways to examine underexplored populations, develop AI that recognizes emotional complexity, and ultimately advance the goal of emotional authenticity in science and society.

## 3. Synthetic Measurement and the Illusion of Emotion Science

The methods we use to measure emotion often define the conclusions we are permitted to draw. If the tools themselves are designed under assumptions of emotional stability, predictability, or universality, then we risk reducing emotion to those forms that comply with those assumptions. This raises a critical question: are we measuring authentic, embodied emotion—or its synthetic shadow?

### 3.1. The Observer Effect in Affective Science

The observer effect—originally formulated in the realm of quantum physics to describe how the act of observation alters the state of a system—is increasingly relevant in psychological and neuroscientific contexts. In affective science, the mere act of measuring emotion can influence the emotion being measured. This is particularly true when the individual becomes aware of being observed, recorded, or evaluated.

Emotional expression is not a neutral signal that simply reflects an internal state. Rather, it is a socially modulated behavior, deeply sensitive to context, perceived surveillance, and the goals of the observer or setting. When participants realize that their emotions are under scrutiny—whether through facial coding systems, psychophysiological sensors, or self-report instruments—they often engage in forms of strategic regulation. This may include amplifying certain feelings, minimizing others, or substituting a more acceptable emotion in place of one they do not feel comfortable expressing [51]. The result is a behavioral and physiological profile that may not correspond to the natural emotional state but instead reflect a synthetic adaptation to the observational context [52].

This dynamic creates a recursive feedback loop between observer and observed. The participant’s behavior changes in anticipation of how it will be interpreted or judged. In research settings, this often manifests as a subtle performance: the participant does not simply experience an emotion—they curate it. This effect is magnified when participants are exposed to visible monitoring technologies such as cameras, EEG caps, thermal sensors, or emotion recognition algorithms. The knowledge that one’s body, brain, or face is being converted into data inevitably alters emotional authenticity. From a computational perspective, this produces contaminated input—data that encode not raw emotion but emotion processed through layers of social filtration and impression management.

In high-stakes contexts, such as forensic evaluations or polygraph examinations, this observer effect becomes even more pronounced. The individual is not just performing for scientific inquiry but for survival—legal, social, or reputational. In these scenarios, emotions like fear, calmness, and surprise may be strategically modulated to avoid suspicion or to project credibility. A calm demeanor may be deliberately constructed through breath control or muscle relaxation, while distress may be suppressed through cognitive reappraisal or emotional detachment. In such cases, the physiological markers typically associated with “truth” or “emotion” are not indicators of spontaneity, but of controlled signaling behavior [53].

Even in laboratory-based emotion research, where the stakes are considerably lower, the presence of structured observation can distort data validity. Participants presented with emotionally charged stimuli—images, narratives, sounds—may still engage in impression management, especially when facial expressions, body posture, or vocal feedback are being recorded. They may attempt to appear emotionally appropriate, resilient, or empathetic, depending on what they assume the experimenter expects or values [54]. In essence, the lab becomes a stage, and emotion becomes a script performed for an invisible audience.

Methodologically, this presents a systems-level challenge: how can we trust emotion data if the act of measurement systematically alters emotional expression? The observer effect introduces a form of noise that is not random but structured—it reflects societal norms, individual socialization histories, and the perceived goals of the observer. This is particularly challenging in the design of emotion-sensitive systems or affective computing models, which often rely on labeled datasets collected under observation. If these systems are trained on data that reflect observer-mediated behavior, their algorithms risk learning not what emotion is, but how it behaves under surveillance.

Ultimately, the observer effect reminds us that emotion is not merely something humans have—it is something we perform, especially when we know we are being watched. As affective science moves increasingly into computational and applied domains, it must grapple with this core epistemological dilemma: what counts as “authentic” emotional data in a world where observation is nearly constant?

It is important to clarify that our critique does not deny the empirical value of EEG in capturing oscillatory markers associated with affective states. Indeed, studies have robustly linked gamma-band synchrony to heightened emotional arousal and attentional engagement [55,56]. However, we argue that these neurophysiological correlates, while statistically significant, are often interpreted within reductionist paradigms that treat them as direct windows into emotional authenticity. Our concern is with the ontological leap—that is, the assumption that oscillatory activity reflects the depth, origin, or personal truth of an emotional state. Emotional authenticity is shaped not only by neural activation but also by context, history, intentionality, and socio-affective scripts. Therefore, we propose a cautious interpretation of EEG data—acknowledging its strengths while resisting its overextension into domains of authenticity or emotional meaning-making.

### 3.2. EEG and the Problem of Affective Surfaces

Electroencephalography (EEG) has long been hailed as a non-invasive window into the “emotional brain.” With its ability to capture millisecond-scale fluctuations in cortical activity, EEG-based emotion detection technologies promise real-time insight into human affective states. These systems have been widely adopted in both research and applied contexts, including neuromarketing, user experience testing, clinical diagnostics, and affective computing. Yet despite their technical sophistication, a fundamental problem remains: EEG primarily measures electrical patterns, not emotions themselves.

At its core, EEG records neural oscillations—patterns of voltage fluctuations across the scalp that correlate with underlying brain activity. These patterns can reflect general states of arousal, attention, fatigue, and cognitive engagement, but they do not offer a direct, one-to-one correspondence with discrete emotional states such as fear, joy, or sadness [57]. Instead, EEG data often capture affective surfaces—transitory manifestations of cognitive–somatic activation that are highly sensitive to task design, environmental context, individual variability, and interpretive frameworks [58]. This makes emotional decoding from EEG as much an inferential task as a technical one.

The challenge intensifies when EEG is used to detect synthetic emotions—that is, emotional responses that are strategically performed or socially conditioned. In many experimental paradigms, participants are presented with emotionally charged stimuli (e.g., images from the International Affective Picture System, emotional words, or videos) and instructed to react. However, these reactions often involve emotional scripts rather than genuine, spontaneous affective experiences. As such, the EEG signal captured reflects not raw emotional generation but a socially shaped performance—a neural correlate of compliance, not authenticity [59].

This has profound implications for both research validity and computational modeling. When synthetic emotions are used as ground truth labels for training affective algorithms, the resulting models may be highly accurate at detecting performative emotion while remaining blind to spontaneous affect. In this scenario, the EEG becomes less a sensor of feeling and more a recorder of rehearsed neural acts—emotional simulations enacted within an experimental frame.

In affective computing, this issue compounds. Machine learning algorithms trained on EEG datasets labeled according to externally validated emotion categories (e.g., “happy,” “angry,” “neutral”) are inherently limited by the quality and authenticity of the training data. If the emotional expressions used in these datasets are scripted, culturally constrained, or distorted by observational awareness, then the bias becomes embedded into the model. What appears as emotion recognition is, in fact, recognition of a narrow emotional vocabulary tied to a specific performance context. These models may generalize poorly across populations, misclassify atypical or neurodivergent emotional expressions, and struggle in open-ended, real-world applications.

Psychologically, this invites scrutiny of surface-level versus experiential emotional measurement. EEG excels in temporal resolution but lacks access to the subjective depth of feelings such as grief, excitement, or longing. Emotional experience is layered, often blending cognitive appraisal, interoceptive feedback, narrative memory, and social context. EEG, by contrast, offers a flattened, externalized snapshot—valuable, but incomplete.

From a computational perspective, this signals the need for multimodal approaches—integrating EEG with other biosignals (e.g., heart rate variability, skin conductance), behavioral data, and contextual metadata. It also calls for probabilistic emotion modeling, where emotional states are treated not as discrete outputs but as dynamic, context-dependent estimations.

While EEG-based tools provide important insights into the brain’s electrical activity during affective episodes, they are constrained by their reliance on observable surface states. When used to detect synthetic emotions, they often reflect performative neural patterns instead of genuine affect. This limitation does not negate the value of EEG—it remains a powerful tool—but underscores the need for more nuanced interpretations of what such signals truly represent. For example, Alahmadi and collaborators [60] argue that inferring emotion from facial expressions alone is fraught with context insensitivity and overgeneralization. Our framework critiques a similar reductionism in measurement tools, suggesting that they risk reinforcing performative emotional norms rather than detecting authentic internal states. Similarly, Li and collaborators [61] point to classification ambiguity in EEG-based emotion recognition systems, which echoes our concern with synthetic misidentification across modalities.

### 3.3. The Polygraph and Emotional Equivocation

The polygraph—often mythologized as a lie detector—continues to occupy a controversial space in both forensic practice and popular imagination. Its underlying premise is simple but powerful: that deception, or emotionally charged internal conflict, will reliably produce physiological signals distinguishable from those of truthfulness or emotional neutrality. Core measurements such as skin conductance (electrodermal activity), heart rate, and respiration are assumed to reflect involuntary autonomic arousal triggered by guilt, anxiety, or internal dissonance. However, this assumption overlooks the complexity, variability, and manipulability of emotional and physiological responses [62].

One of the central challenges with polygraphy is that physiological activation is not exclusive to deception. Elevated heart rate or increased sweat gland activity can just as easily signal anticipatory anxiety, environmental discomfort, trauma-related arousal, or high self-monitoring in individuals concerned about being misread. Moreover, individual differences in emotional regulation styles—such as suppression, reappraisal, or dissociation—can significantly distort polygraph readouts. Some people display minimal autonomic reactivity even under stress, while others experience significant arousal even when telling the truth. This leads to false positives (where innocent individuals appear deceptive) or false negatives (where deceptive individuals exhibit controlled physiological responses) [63].

In this light, the polygraph functions less as a measure of emotion itself and more as an index of emotional ambivalence under pressure—ambiguous states of heightened self-regulation, vigilance, and stress that arise under the weight of observation, accusation, or threat. What is being measured, therefore, is not the presence or absence of guilt or honesty, but the behavioral and physiological choreography that individuals develop to survive scrutiny. Emotional expressions in this context become strategic outputs, shaped by learned associations between visibility and consequence [64].

From a psychological standpoint, this means the polygraph readouts are context-sensitive, shaped by power dynamics, individual history, and emotional resilience. A trauma survivor may show physiological signs of arousal when asked about past events—not because they are lying, but because the context evokes unresolved emotional memory. Similarly, individuals with high social anxiety or obsessive self-monitoring may exhibit elevated arousal during polygraph questioning simply due to fear of being misunderstood.

While traditional polygraphy interprets autonomic arousal (e.g., skin conductance, respiration, blood pressure) as indicators of deception or affective intensity, we reinterpret such data as reflecting emotional equivocation—the internal dissonance between experienced and performed affect. To move beyond conceptual critique, we propose a testable framework grounded in affective neuroscience. Specifically, we suggest that equivocation may involve increased engagement of neural circuits associated with conflict monitoring, such as the anterior cingulate cortex (ACC), which has been implicated in emotional conflict, ambiguity, and cognitive–emotional regulation [65].

Experimental designs comparing polygraph responses during authentic, suppressed, and simulated emotional states, in conjunction with neurophysiological data, could offer measurable criteria to distinguish equivocation from genuine arousal. Such integrative approaches may allow for the operationalization of emotional equivocation as a distinct physiological and cognitive profile.

From a computer science and systems modeling perspective, the polygraph highlights the broader problem of ambiguous input interpretation. Physiological signals such as galvanic skin response or respiration rate are inherently non-specific—they are affective but not diagnostic. When machine learning models are trained on such signals without rich contextualization or multi-layered inference mechanisms, they risk producing misleading classifications. Just as the polygraph may mistake nervousness for deceit, emotion recognition systems may mistake controlled affect for emotional emptiness, or expressive variance for instability.

In environments where the stakes are high—legal interrogations, immigration interviews, or national security screenings—this misattribution can lead to real-world harm: wrongful accusations, biased judgments, and psychological distress. These consequences are not simply technical failures; they reflect a deeper epistemological error—the belief that emotion, in its truth, can be extracted from the body without regard to context, meaning, or relational dynamics.

To address this, both psychological practice and computational design must move beyond signal reductionism. Emotions are not singular events encoded in discrete spikes of arousal; they are temporally distributed, meaning-laden, and socially informed processes. A machine—or a human interrogator—that interprets physiological data without understanding the broader human context is not reading emotion; it is decoding reactivity. And reactivity, as the polygraph shows, is never emotionally neutral.

### 3.4. Psychometric Illusions: Self-Reports and Social Scripts

Self-report instruments are foundational tools in psychological science, widely used to assess emotional tendencies, mood states, personality traits, values, and cognitive schemas. Their appeal lies in their accessibility, efficiency, and the assumption that individuals can reliably introspect and report on their inner lives. However, the nature of self-report data is far from neutral. These instruments are grounded in linguistic structures and cultural conventions, meaning they often reflect how people think they feel—or how they wish to be seen—rather than capturing spontaneous, embodied emotional experience [66].

When individuals complete items such as “I enjoy helping others” or “I often feel sad,” they are not accessing raw affective states in the present moment. Instead, they are drawing on semantic memory—the part of long-term memory that stores factual knowledge about the world and the self. Respondents interpret the meaning of the question, retrieve self-relevant knowledge, and select a response that best approximates their self-understanding [67]. In doing so, they rely on self-schemas—cognitive frameworks that organize and structure self-related knowledge—and their moral self-concept, which guides value-laden judgments and socially sensitive self-disclosure.

This process is far from emotionally neutral. Self-reports are shaped by cognitive filtering, social desirability, and, often, idealized schemas—mental representations of how individuals aspire to be seen, both by themselves and by others. While semantic memory and self-schemas offer structure, idealized self-views may inflect how respondents position themselves within the questionnaire. For example, a person may report being generous, resilient, or emotionally intelligent not solely because these traits reflect lived experience, but because they resonate with internalized ideals or socially preferred identities. These subtle shifts introduce synthetic emotional data into psychological measurement—filtered not through deception, but through culturally constructed scripts and self-representational motives [68].

Furthermore, many psychometric instruments, particularly those used in large-scale personality assessment or schema evaluation, rely on simplified, binary, or Likert-based response formats. They often reduce emotion to forced dichotomies: happy vs. sad, anxious vs. calm, or introverted vs. extroverted. This categorical compression limits the ability to capture emotional nuance—especially ambivalence, contradiction, or mixed feelings, which are common in real-life affective experience. Emotions are rarely cleanly separated; they are often layered, dynamic, and contextually entangled. However, when measured via self-report, these complexities are flattened for ease of analysis and standardization [69].

Such flattening may be particularly problematic in populations whose affective and cognitive patterns diverge from dominant cultural scripts. Neurodivergent individuals, trauma survivors, and those from non-Western or collectivist cultures may experience and express emotion in ways that do not align with the assumptions embedded in standard psychometric tools. For example, emotional masking in autistic individuals or hypervigilance in trauma survivors may result in questionnaire responses that underreport distress or misrepresent emotional style—not due to lack of insight, but because the questions fail to accommodate non-normative affective architectures [70].

From a systems and computational perspective, self-report instruments generate structured data, but the structure is imposed, not discovered. These instruments rely on input that is pre-encoded through language, filtered through cognition, and shaped by context. Machine learning models trained on such data may produce elegant patterns, but they are often trained on narratives, not feelings—on what people say about themselves, not on what they feel in real time. This has implications for everything from diagnostic classification to affective recommender systems and emotion-aware AI, where systems may replicate the same blind spots inherent in their data sources.

Ultimately, while self-report questionnaires remain valuable tools for psychological exploration and clinical screening, their epistemic limitations must be foregrounded. They do not measure emotion directly; they measure the representation of emotion as mediated by memory, language, social context, and idealized identity. To mitigate these distortions, researchers and clinicians must contextualize self-report data within a broader assessment framework—combining it with behavioral observation, physiological markers, narrative interviews, and ecological sampling.

Thus, self-report offers a window not into raw emotional truth, but into the stories people tell about their emotions, guided by what they know, what they remember, and what they are willing to share.

### 3.5. Toward Ecologically Valid Measures of Emotion

Efforts to understand and measure human emotion have historically relied on simplification: isolating variables, controlling environments, and categorizing experiences into discrete emotional states. While this reductionist approach offers clarity and replicability, it risks stripping emotion of its context, spontaneity, and fluidity. In order to capture natural emotional states—those not shaped by external expectations or artificial elicitation—emotion science must shift toward ecologically valid methods that prioritize authenticity over standardization.

Natural emotion does not always conform to fixed temporal windows, externally defined categories, or laboratory stimuli. It often arises in unpredictable ways, embedded in daily life and shaped by an individual’s relational, cultural, and biographical contexts. To move closer to capturing this complexity, measurement must become less invasive, less categorical, and more temporally sensitive. Instead of relying on forced-choice items or time-locked experimental triggers, researchers can draw on longitudinal journaling, passive biosensor tracking, and qualitative narrative elicitation—approaches that allow emotional expression to emerge organically over time, within real environments [71].

These methods acknowledge that emotion is not always loud, expressive, or externally legible. Much of what constitutes authentic emotion is internal, quiet, and nonlinear—a physiological tightening, a recurring thought, a relational shift. Emotion, in this view, is not a snapshot but a process, one that unfolds across multiple timescales and channels. Recognizing this, researchers must design tools and methodologies that can detect emotional nuance without forcing it into rigid frames.

Critically, emotional responses must also be contextualized within the individual’s broader ecosystem: their relational histories, cultural norms, neurodiversity profile, and prior emotional conditioning. The same physiological signal—such as elevated heart rate or facial tension—can mean vastly different things depending on the situation: excitement, anxiety, reactivity, trauma, or even learned suppression. Without this context, affective data become flat—technically rich but psychologically hollow [72].

Recent methodological advances offer promising avenues for advancing emotional measurement. For instance, interoceptive accuracy tasks aim to assess how well individuals perceive their internal bodily states, such as heartbeat or breathing rates—key dimensions of emotional awareness. Momentary ecological sampling collects self-reports in real-time, through mobile devices, allowing emotion to be captured as it is experienced, rather than reconstructed after the fact. And hyper-scanning techniques, which measure neural synchronization between individuals, offer powerful insight into affective resonance in social interaction—shifting the unit of analysis from the isolated brain to the relational dyad [73,74].

Together, these approaches represent a movement toward emotion-as-process rather than emotion-as-output. They treat emotional expression not as a discrete signal to be extracted, but as an emergent phenomenon embedded in systems of meaning, memory, and embodiment. From a computational perspective, this shift suggests the need for adaptive, probabilistic, and context-aware models—systems that can infer affective states not from isolated data points, but from patterns distributed across time, space, and modality.

This approach also demands humility. The tools we use to study emotion are not neutral—they shape what we are able to see. If our instruments are built to detect strong expressions, binary states, or time-locked reactions, then we will find only what we are equipped to measure. Subtle, conflicted, or contextually embedded emotions may remain invisible, not because they do not exist, but because they resist capture by dominant methods.

The illusion of objectivity in emotion science stems not from a lack of rigor, but from a mismatch between the complexity of emotional life and the simplifications imposed by our tools. To overcome this limitation, researchers and system designers must interrogate their assumptions, recalibrate their frameworks, and develop tools that are not only precise, but respectful of the messy, contradictory, and beautifully human texture of real emotional life.

## 4. The Role of AI, Neurodiversity, and Emotional Authenticity

The proliferation of artificial intelligence technologies—particularly in affective computing and human–machine interaction—demands a critical re-examination of what it means to recognize, simulate, or generate emotion. At the same time, emerging research on neurodiversity highlights the variability of emotional expression, challenging assumptions of what “authentic” emotion looks like. This section examines how synthetic emotion manifests within AI systems and human social systems, with special attention to neurodivergent populations as potential exemplars of natural emotional processing.

### 4.1. Affective AI and the Replication of Synthetic Emotion

Affective computing—systems designed to detect, interpret, and sometimes simulate human emotions—has emerged as one of the most visible frontiers in artificial intelligence. These technologies span a wide range of applications, from facial expression recognition and sentiment classification to EEG-based affective feedback systems. The central promise is compelling: that machines can understand human emotion in real time and adjust their responses accordingly. However, this promise rests on a critical and often unexamined foundation: training data derived from human behavior that are themselves deeply synthetic [75].

Human emotion, especially in observable form, is rarely expressed in a vacuum. The facial expressions, vocal tones, and gestures used to train affective AI are almost always context-dependent, socially modulated, and strategically constructed—that is, they reflect emotional performance more than raw affect. As a result, AI models do not learn to detect emotion in the deep psychological sense, but rather the outward signs of emotion as constrained by cultural scripts and social expectations [76].

In practice, this means that affective AI systems are optimized to identify stereotyped, legible emotional cues—the smile that signifies happiness, the furrowed brow that suggests concern, or the elevated voice pitch associated with excitement. These mappings, while computationally efficient, reinforce a narrow and often culturally biased set of emotional archetypes [77]. Emotional expressions that fall outside these templates—because they are subtle, culturally nuanced, idiosyncratic, or neurodivergent—may be misclassified, ignored, or penalized by the system.

The implications of this are profound. Rather than deepening our understanding of human affect, affective AI may reify a model of emotion that is already distorted—one that privileges external conformity over internal authenticity [78]. This creates a feedback loop in which human users, aware that machines are interpreting their emotions, may begin to perform in ways that are more easily “read” by the system, thus reinforcing the very synthetic patterns the technology was meant to illuminate.

Ironically, as AI systems become more “emotionally intelligent,” they increasingly mirror the same limitations, biases, and simplifications that have long plagued human emotion research. Systems designed to detect empathy, sincerity, or deception, for example, often focus on identifying the most legible cues—those that are easiest to interpret or quantify—rather than probing the authenticity or complexity of the underlying emotional state [79].

This preference for legibility over authenticity is not a technical glitch but a structural limitation. Machine learning algorithms rely on clearly labeled data and observable features. In doing so, they are inherently biased toward emotional expressions that are externalized, codified, and consistent. Individuals who are especially skilled at displaying emotion—whether through learned social behavior, professional training (e.g., actors, caregivers), or simply high expressive fluency—are often “read” as more authentic, even if their internal state diverges from the performance. Meanwhile, those who express emotion in less conventional or more restrained ways—due to culture, personality, trauma, or neurodivergence—may be misread as emotionally flat, deceptive, or insincere.

This becomes particularly problematic in sensitive domains such as law enforcement, healthcare, education, and hiring, where AI-driven emotional insights are increasingly used to inform real-world decisions. A system might falsely flag a nervous but truthful individual as deceptive or underestimate a patient’s distress because their face lacks the expected markers of pain. These false interpretations are not just errors—they are ethical liabilities with real human consequences.

Another critical limitation lies in the absence of contextual understanding. Emotion does not exist in a vacuum; it is shaped by individual history, social setting, cultural norms, and moment-to-moment cognitive processing. An elevated heart rate might signify fear in one person, excitement in another, or simply physical exertion. Facial expressions may be consciously masked or socially exaggerated. Without access to this surrounding context, AI systems are left to infer emotional meaning from superficial features alone—features that may or may not reflect the individual’s actual state.

To move beyond this limitation, affective AI must evolve from cue detection to meaning inference. This shift requires models that are not only trained on diverse datasets but are also designed to handle ambiguity, uncertainty, and nuance. Probabilistic modeling, temporal analysis, multimodal fusion, and attention to intra-individual variability can all help bridge the gap between surface behavior and inner experience.

Affective AI—when built upon synthetic expressions—risks learning not what it means to be human, but what it means to perform humanity. While these systems can be useful for certain applications, their current structure favors clarity over complexity, performance over presence. As we continue to develop emotionally aware machines, we must also develop a critical awareness of what, exactly, those machines are learning to recognize—and what they are likely to miss.

While this critique emphasizes structural and epistemological concerns about emotional authenticity in affective AI, it is important to acknowledge the emergence of technical solutions aimed at mitigating dataset bias. Approaches such as adversarial debiasing [80], uncertainty-aware sampling [81], and fairness-oriented uncertainty estimation [82] represent meaningful advances in refining model robustness and fairness. Additionally, efforts toward explainable affective computing [83] and ethical audits for emotion recognition systems [84] suggest a growing sensitivity to these concerns within the field.

Nonetheless, our argument is not that these techniques are unavailable—but that they are often insufficient when foundational assumptions about emotion remain unexamined. Even when models are technically debiased, they frequently inherit ontologies that treat emotion as universally legible and reducible to surface features. Moreover, the reliance on synthetic datasets to simulate rare or ambiguous emotional expressions, although methodologically useful [85,86], can introduce structural distortions if these datasets reinforce dominant emotional grammars [87]. We propose that mitigating bias in affective AI must involve both algorithmic innovation and conceptual reform—where emotion is treated not merely as a classifiable input but as a socioculturally embedded and contextually expressed phenomenon.

### 4.2. Synthetic Emotion as a Prerequisite of Social Functioning

In contemporary society, the performance of emotion is not merely expected—it is required. Synthetic emotion, understood as emotionally regulated expression shaped to meet social, institutional, or cultural expectations, plays a central role in enabling individuals to function across a wide range of interpersonal and professional contexts. Emotional regulation, norm compliance, and situational adaptation form a triad of social–emotional competencies that are not just encouraged but often demanded as conditions for belonging, employment, and relationship stability [88].

From early development, children are taught which emotional displays are socially appropriate—when to smile, when to apologize, when to suppress anger, and how to present politeness even in discomfort. These behaviors are not inherently dishonest; rather, they are instrumental in facilitating cooperation, reducing social friction, and navigating complex relational hierarchies. Over time, these emotional scripts become internalized, forming cognitive–emotional habits that guide interpersonal behavior well into adulthood.

Emotional regulation involves the ability to manage and modulate affective responses in ways that are considered socially appropriate. This regulation allows individuals to avoid impulsive reactions, de-escalate conflicts, and maintain composure in emotionally charged environments. It supports resilience, improves social outcomes, and enhances perceived maturity and professionalism.

Norm compliance refers to adherence to explicit and implicit social rules, which dictate how emotion should be expressed—or not expressed—in particular contexts. Whether in a courtroom, classroom, or workplace, individuals are expected to present emotions in line with setting-specific expectations. Failure to conform may result in social sanctions, alienation, or misjudgment.

Situational adaptation, meanwhile, is the capacity to flexibly modify emotional expression based on the demands of a given social environment. The same individual might adopt distinct emotional personas when interacting with a romantic partner, a supervisor, or a customer. This adaptive flexibility is not only rewarded but often essential for success in socially complex roles.

Together, these abilities form the foundation of synthetic emotional competence—the capacity to navigate social life through the calculated display or suppression of emotion. While this competence can facilitate relational harmony and workplace effectiveness, it also reflects a societal infrastructure that prioritizes emotional predictability over emotional authenticity. Emotion is often judged not by its subjective origin but by its external presentation: is it appropriate, stable, intelligible?

This emphasis on conformity and control comes with significant psychological costs. When emotional expression is persistently shaped to meet external expectations—rather than arising from internal states—individuals may experience chronic emotional masking. This phenomenon involves the repeated suppression, alteration, or substitution of one’s genuine affective responses to conform to perceived social norms. Over time, masking can result in a progressive detachment from one’s natural emotional experience [89].

The consequences of long-term masking are profound and multifaceted:

- Alienation emerges when individuals feel unable to express their true emotional selves, leading to difficulty in forming deep, authentic relationships. The emotional persona they project becomes disconnected from their internal experience, and social interactions, while smooth, may feel hollow or performative.
- Exhaustion results from the cognitive and emotional effort required to constantly monitor, adjust, and perform socially acceptable emotion. The mental energy spent on self-monitoring can lead to emotional fatigue, burnout, and physical stress-related symptoms, particularly in roles requiring frequent emotional labor.
- Affective dissonance occurs when there is a persistent mismatch between felt emotion and expressed emotion. This internal incongruence can foster a sense of inauthenticity, anxiety, and emotional confusion. Over time, individuals may lose access to their true emotional states, struggling to identify or trust what they genuinely feel [90].

This internal cost is often hidden beneath a socially rewarded exterior. In many environments, synthetically consistent emotion—that is, emotion that is stable, context-appropriate, and aligned with normative expectations—is treated as the benchmark of emotional intelligence, maturity, and psychological health. Those who deviate from these norms may be pathologized or marginalized. Individuals who cry too easily, show little expression, or react with disproportionate intensity may be labeled as emotionally unstable, unfeeling, or socially inept—not because they lack emotion, but because they fail to present it in the socially sanctioned format.

In clinical contexts, this dynamic can be especially problematic. Emotional flatness in neurodivergent individuals, for example, is often misread as apathy or lack of empathy. Conversely, individuals from emotionally expressive cultures may be perceived as histrionic or dysregulated when their affect does not conform to clinical expectations rooted in Western affective norms.

From a systems design perspective, these observations have important implications for how we build and train AI systems intended to interpret human emotion. If machines are calibrated to detect and respond only to emotionally normative behaviors—those that are stable, visible, and legible—then entire populations may be systematically misunderstood or excluded. The AI would not be biased merely because of its algorithmic architecture, but because it is mirroring and reinforcing a human social order built around synthetic emotional norms.

While synthetic emotion facilitates social coordination and institutional functioning, it also risks reducing emotional life to a series of performance scripts. The very skills that promote social integration—regulation, compliance, and adaptation—can, when overused, erode emotional authenticity and wellbeing. To address this tension, we must develop both cultural and technological systems that recognize the psychological cost of emotional conformity and make space for more diverse, contextually grounded expressions of affect.

### 4.3. Neurodiversity and the Visibility of Natural Emotion

Neurodivergent individuals—particularly those with autism spectrum conditions, ADHD, and related cognitive profiles—often engage with emotional life in ways that diverge from mainstream expectations. This divergence is not simply behavioral; it challenges foundational assumptions about what emotion is, how it should be expressed, and who is permitted to define emotional normalcy. Within neurodivergent populations, the gap between felt emotion and socially legible expression is often wide—and misunderstood [91].

Many individuals on the autism spectrum, for example, describe experiencing emotions with great intensity, but simultaneously struggle to convey those feelings in ways that align with neurotypical display rules. This internal–external disconnect is frequently interpreted as emotional flatness, detachment, or even lack of empathy. Yet such interpretations reflect the bias of neurotypical emotional coding—the assumption that emotional authenticity is necessarily expressed through standardized behavioral cues such as facial expression, tone of voice, or physical proximity [92].

The problem is not a lack of emotion, but rather a misalignment between emotional experience and its expected social representation. Emotional expression among neurodivergent individuals may appear muted, idiosyncratic, asynchronous, or nonlinear. These patterns are often pathologized in clinical contexts, leading to diagnostic misunderstandings or social marginalization. Yet this same divergence may be precisely what makes neurodivergent emotionality more authentic—less bound by performance scripts, and more directly connected to internal affective states.

In fact, what is frequently described as an “emotional deficit” in neurodivergent individuals may actually be a rejection—conscious or unconscious—of synthetic emotional norms. Many neurodivergent people do not instinctively engage in emotional masking or social mimicry, not because they are incapable, but because these behaviors may feel unnatural, cognitively taxing, or ethically disingenuous. This relative resistance to emotional scripting—whether deliberate or neurobiologically mediated—reveals a deeper insight: that much of what passes for normal emotional expression in neurotypical society is itself highly constructed [93].

In this way, neurodivergent expression acts as a kind of emotional counter-narrative. It disrupts the assumption that appropriate emotion is synonymous with visible affect. It confronts the cultural privileging of emotional fluency, stability, and predictability—values that, while socially rewarded, may obscure the underlying variability and depth of genuine emotional life.

From a psychological perspective, this has significant implications for therapy, education, and diagnostic practice. Emotional intelligence, as commonly defined, is often biased toward outward regulation and interpersonal signaling. Neurodivergent individuals who do not conform to these expectations may be unfairly assessed as lacking insight or empathy, when in fact their emotional processes are simply less performative and more internally centered. Instead of pathologizing difference, clinicians and educators must develop frameworks that recognize and validate diverse emotional trajectories.

From a computational and design standpoint, these insights challenge the assumptions behind affective AI. Systems trained on standardized emotional cues will likely struggle to recognize—or worse, misclassify—neurodivergent emotional expressions. A flat affect may be flagged as indifference; intense eye contact avoidance may be interpreted as deception. These interpretations do not reflect malicious design, but algorithmic alignment with biased emotional training sets. If AI systems are to be inclusive and contextually intelligent, they must be trained to recognize that emotional authenticity is not always behaviorally obvious—and that legibility is not the same as truth.

Neurodivergent expression reveals a central paradox in affective science: that emotional authenticity is not always legible, and emotional legibility is not always authentic. In a world that increasingly rewards emotional polish and performative empathy, neurodivergent individuals may, ironically, offer one of the clearest views into what unfiltered emotional truth actually looks like.

### 4.4. Toward Emotional Authenticity in Human and Machine Systems

Recognizing that synthetic emotion has become the default currency of many social interactions—particularly in professional, mediated, or high-stakes contexts—does not inherently condemn its use. Strategic emotional display is, in many cases, a survival strategy: a form of adaptive intelligence that allows individuals to navigate complex social hierarchies, avoid harm, or meet role-based expectations. The key issue, however, is not the mere existence of synthetic emotion, but the degree to which individuals and systems are aware of its synthetic nature.

In other words, the danger is not in emotional performance per se, but in mistaking performance for truth—a conflation that can lead to misjudgments in clinical diagnosis, interpersonal trust, machine learning systems, and even self-perception. Emotional authenticity, then, is not about purity or spontaneity alone, but about recognizing the layered construction of emotion and the limits of legibility in both humans and machines [94].

From an affective computing standpoint, this awareness calls for a paradigm shift. Current AI systems designed for emotion recognition tend to focus on surface-level features—facial expressions, vocal tone, gesture dynamics, and physiological data. These features are then mapped onto fixed emotional categories using supervised learning techniques trained on static, labeled datasets. But these datasets often reflect staged, socially scripted, or culturally biased displays, rather than spontaneous, context-rich emotional events. In doing so, such systems risk encoding and amplifying the very synthetic patterns they aim to decode.

A more ethically and scientifically grounded approach would involve the integration of probabilistic reasoning, multimodal temporal modeling, and narrative-based frameworks. Rather than treating emotion as a static classification task (“Is this person happy or sad?”), affective AI should approach emotion as a dynamic, context-sensitive signal that may include uncertainty, ambiguity, contradiction, or silence. For instance, a system could flag not just the presence of emotional cues but also the confidence level of interpretation, the contextual constraints, and even the possibility of emotional masking or simulation.

Parallel challenges exist within human systems—particularly in education, psychotherapy, healthcare, and workplace management. There remains a persistent cultural bias that equates appropriate emotional expression with emotional health. Individuals who fail to demonstrate legible affect—whether due to neurodivergence, trauma, cultural difference, or introversion—are often misunderstood, pathologized, or dismissed. Conversely, those who have mastered the choreography of synthetic emotion may be overestimated in terms of empathy, engagement, or sincerity [95].

To correct these distortions, human-facing systems must be trained to distinguish emotional presence from emotional appearance. This requires not only technical skill, but also ethical humility and interpersonal sensitivity. Therapists, educators, managers, and AI designers alike need tools that support deeper attunement to internal states, rather than reliance on behavioral scripts. This includes tolerating emotional ambiguity, accepting non-normative affective styles, and resisting the impulse to collapse complex emotional lives into binary categories.

Ultimately, the path forward involves a rebalancing of our attention—shifting focus away from what is most visible and toward what is most authentic. In both machine and human systems, the goal should not be to perfect emotional performance, but to create spaces in which natural emotion can safely emerge. Emotional authenticity cannot be programmed into an algorithm or enforced through institutional policy—but it can be nurtured, safeguarded, and recognized.

This means building systems—technical and social—that are comfortable with silence, complexity, and delay. It means embracing emotion as a process, not just a state. And it means acknowledging that sometimes, the most meaningful emotions are those that are hardest to detect, quantify, or explain—but that nevertheless shape the very core of our human experience [96].

### 4.5. Emotional Evolution or Emotional Devolution?

The historical arc of human emotion is not static; it has always evolved in dialogue with the social, technological, and ecological environment. Yet the current age introduces an unprecedented paradox: while emotional visibility is at an all-time high—broadcast through social media, quantified by apps, and mined by algorithms—emotional authenticity may be in decline. This calls attention to a profound question: are we evolving or devolving emotionally?

As human societies have grown more interconnected and technologically mediated, the space for spontaneous, unfiltered emotional expression has narrowed. Emotional regulation is no longer merely interpersonal—it has become algorithmically optimized, culturally surveilled, and globally exposed. Children learn early how to “perform” feelings in ways that meet digital expectations. Professionals are increasingly evaluated not only on performance outcomes but on affective tone, relational demeanor, and visible positivity. In such a world, emotional display becomes a form of capital, and the line between sincerity and strategy becomes ever more difficult to draw [97].

This trend might be interpreted as an adaptive evolution—one in which emotional performativity becomes a survival skill, honed through centuries of social learning and now refined through digital feedback loops. From this view, emotional “devolution” is not regression but optimization for a new context: the context of surveillance, simulation, and social branding.

But this transformation carries psychological costs. The more we optimize emotion for display, the more we risk losing connection with its intrinsic function—to signal internal states, form bonds, and guide behavior based on authentic inner experience. A society that prioritizes visibility over vulnerability runs the risk of producing emotionally fragmented individuals: outwardly expressive, inwardly disconnected.

And yet, paradoxically, this very saturation of synthetic emotion may trigger a corrective pushback. In the face of emotional commodification, individuals increasingly seek moments of authenticity—spaces where affect can emerge unshaped, unbranded, and unmeasured. We see this in the growing appeal of trauma-informed therapy, in the validation of neurodivergent expression, and in the design of AI systems that are not only affective but affect-aware—designed to grapple with the limitations and moral implications of interpreting human emotion [98].

This tension between control and emergence, between simulation and truth, mirrors a broader evolutionary dialectic. Perhaps the future of emotional life lies not in rejecting synthetic emotion, but in becoming more critically aware of its role—integrating it consciously rather than unconsciously performing it. A truly evolved emotional system may be one that can discern when to perform, when to withhold, and when to surrender to raw, unscripted feeling.

Emotion, at its most elemental, refuses full capture. It resists total definition, evades precise measurement, and often arises in contradiction to the roles we inhabit. This irreducibility is not a flaw—it is the core of what makes emotion meaningful. Even in a world of deepfakes, AI-generated empathy, and curated selfhood, emotion remains one of the last frontiers of the real [99].

## 5. Conclusions: Redefining Emotion for the Post-Synthetic Age

This paper proposed a five-type typology of emotional expression—transparent, camouflaged, synthetic, contaminated, and residual emotion—as a conceptual framework for understanding the layered complexity of affective life in contemporary contexts. This typology serves as a foundational step in disentangling authentic from socially shaped or strategically performed emotional responses. By integrating this model with a critical review of measurement paradigms and the influence of contextual and sociocultural factors, we argue for a recalibration of how emotion is conceptualized, detected, and interpreted across disciplines. Our intention is not to replace existing emotion theories, but to expand their scope by offering a vocabulary that reflects emotional nuance, ambiguity, and fluidity—particularly in light of evolving technologies, neurodiversity, and lived experience.

Emotion, once considered the spontaneous language of the inner self, has increasingly become a coded dialect of social performance. This paper has argued that much of what we currently classify and quantify as “emotion” may be more synthetic than natural—filtered through layers of social learning, expectation, surveillance, and technological modeling. These synthetic emotional displays serve adaptive, relational, and cultural purposes, but they rarely represent the full depth and spontaneity of affective life.

Recent studies in affective neuroscience and psychological science confirm the variability and social modulation of emotion, especially in technologically mediated contexts. For instance, Pang and collaborators [100] and Fang and collaborators [101] emphasize that facial expressions and vocal cues are often ambiguous and culturally shaped, challenging their use as reliable emotional indicators. Similarly, Gkintoni and collaborators [102] note the limitations of EEG-based emotion recognition systems, particularly when applied without attention to context and individual variability. Our typology contributes to this growing body of work by providing a conceptual language to differentiate types of expression that may appear similar but are functionally distinct.

The issue, however, is not the existence of synthetic emotion—it is the uncritical elevation of synthetic emotion as the standard. When science builds its instruments, when clinicians make diagnoses, or when AI is trained to “read” emotion, these practices often treat observable affect as inherently truthful. Yet as we have seen, such expressions may reflect rehearsed scripts, defensive adaptations, or normative pressures rather than internal states. The consequences of this confusion ripple across disciplines—from misclassification in psychometrics to misinterpretation in courtroom lie detection, from oversimplified AI emotional models to educational systems that reward emotional conformity over authenticity.

Importantly, the divide between natural and synthetic emotion is not abstract or pedantic. It is felt most acutely by those who cannot easily conform to the dominant emotional grammar: neurodivergent individuals who emote differently, trauma survivors who regulate through suppression, or cultural minorities whose expressive norms are not legible to the majority gaze. These groups are often marginalized not because of what they feel, but because of how they express—or fail to express—what they feel. The emotional data collected about them are frequently incomplete, distorted, or pathologized.

Thus, redefining emotion for the post-synthetic age is not simply a call for better instruments—it is a call for epistemic humility, for systems that listen as much as they detect and that hold space for ambiguity as much as they seek classification. We encourage researchers to adopt reflexive practices, including those outlined by Finlay [103] and Pillow [104], which emphasize critical self-awareness and the socio-affective positioning of the researcher.

For this redefinition to take root, emotion science must evolve in five critical directions:

1. Clarify conceptual boundaries: The field must distinguish carefully between affect (raw, often preconscious physiological responses), emotion (socially shaped and cognitively interpreted patterns), and feeling (subjective experience). Without these distinctions, research becomes a blur of overlapping constructs, reducing emotional phenomena to overly broad categories.
2. Diversify emotional typologies: The standard happy–sad–angry taxonomies no longer suffice. Human emotion is frequently hybrid, ambivalent, masked, or residual. Typologies must reflect this multiplicity, acknowledging that emotional states often coexist in paradoxical forms.
3. Integrate context-sensitive methods: Tools like EEG, polygraphs, and facial analysis provide data but not always meaning. Measurement systems must evolve to account for lived context—social histories, cultural norms, relational dynamics, and internal scripts—so that emotion is not treated as an isolated signal but as an embedded narrative.
4. Recognize neurodivergent expression as valid: Emotional authenticity cannot be judged solely by its resemblance to neurotypical behavior. A flat affect, averted gaze, or absence of verbalized feeling do not mean absence of emotion. Scientific and clinical systems must affirm alternative expressive styles as legitimate and intelligible in their own right.
5. Model emotion in AI systems with humility: Artificial intelligence should not aspire to replicate human emotional behavior as if it were a fixed code. Instead, it should be trained to navigate emotional uncertainty—to recognize when data are ambiguous, when silence is meaningful, and when affect defies legibility. Emotional intelligence in machines must begin with emotional modesty.

Beyond theory, these insights have immediate applicability for educational settings, media production, and the development of affective technologies. In education, for instance, fostering emotional literacy should move beyond emotional labeling and instead incorporate reflective practices that help students identify camouflaged or residual emotions shaped by school culture. Media professionals and designers of emotion-recognition tools should avoid overreliance on expressive congruence and instead develop guidelines that account for social masking, trauma-related suppression, or cultural expressivity norms. Furthermore, educational policy must accommodate emotional pluralism—by integrating training for teachers to recognize diverse emotional signals and by updating curricula to include emotional complexity as a pedagogical asset rather than a deviation from normativity.

In the post-synthetic age, emotional literacy will no longer be measured by how well one performs feeling, but by how deeply one understands its origin, modulation, and unmediated affect. True emotion is neither a performance nor a dataset—it is a process, often contradictory and elusive, that defies simplification. It is not something we display, but something we live.

And because emotion will always resist full capture—by machine, by scale, by algorithm, or even by language—it demands from us not just analysis but respect. If science and society can shift from chasing emotional clarity to honoring emotional complexity, we may yet build a culture—and a technology—that recognizes not just what people feel, but how, why, and in whose voice they express it [105,106].

This article makes a threefold contribution: theoretically, by introducing a novel typology that redefines how we classify emotional expressions beyond traditional emotion categories; methodologically, by critiquing the assumptions underlying common measurement tools in affective science and AI; and practically, by offering concrete directions for emotional literacy, educational policy, and technology design. It adds to a growing interdisciplinary discourse calling for epistemic humility, socio-contextual interpretation, and ethical sensitivity in the study and application of human emotion.

## Figures and Tables

**Table 1:** The refined typology of emotion.

Emotion Type	Definition	Example
Transparent Emotion	A directly experienced and spontaneously expressed emotional state, where there is minimal or no filtering, inhibition, or distortion between internal affect and external behavior. This form of emotion often appears in contexts of safety, intimacy, or immediate reaction.	A child cries loudly and reaches for a parent after being startled by a loud noise, openly displaying fear.
Camouflaged Emotion	An internal emotional experience that is consciously or unconsciously masked, suppressed, or redirected, typically in response to social norms, power dynamics, or perceived risks of emotional exposure. The emotion is present but not externally observable.	An employee feels humiliated during a team meeting but maintains a composed facial expression to preserve professionalism.
Synthetic Emotion	A deliberately constructed or socially adapted emotional expression that may not correspond to the individual’s true affective state. These emotions are often rehearsed or habitual responses to social expectations or institutional roles.	A customer service representative offers an enthusiastic greeting and smile despite feeling emotionally detached or fatigued.
Contaminated Emotion	An emotion that originates authentically but becomes gradually altered, distorted, or confused through repeated social invalidation, trauma, suppression, or internal conflict. The emotional signal is no longer clear or trustworthy to the self.	A person who once felt genuine excitement about personal achievements now experiences guilt or anxiety when praised, due to past experiences of being criticized for showing pride.
Residual Emotion	An emotional state that continues to exist well beyond the original triggering event, often maintained by cognitive–emotional loops such as rumination, unresolved trauma, identity narratives, or embodied memory. It reflects the temporal persistence of affect.	A person who experienced betrayal in a past relationship continues to feel distrust and sadness in new, unrelated social interactions.

## Data Availability

No new data were created or analyzed in this study.
